# Primary pancreatic signet ring cell carcinoma: molecular mechanisms and advances in clinical diagnosis and treatment

**DOI:** 10.1186/s12957-025-04106-4

**Published:** 2025-11-27

**Authors:** Jie Zhang, He Huang, Bing Tang, Xiaoli Hu, Zhiqiang Ming, Dan Wan, Qianping Qu

**Affiliations:** 1 Department of Hepatobiliary and Pancreatic Surgery, Zigong First People’s Hospital, Zigong, Sichuan China; 2 Department of Ultrasound, Zigong First People’s Hospital, Zigong, Sichuan China; 3 Department of Radiology, Zigong First People’s Hospital, Zigong, Sichuan China; 4 Department of Pathology, Zigong First People’s Hospital, Zigong, Sichuan China

**Keywords:** Primary pancreatic signet ring cell carcinoma, Molecular mechanisms, Diagnostic strategies, Therapeutic advances

## Abstract

**Purpose:**

To comprehensively integrate current evidence on the molecular mechanisms, diagnostic approaches, and treatment options for primary pancreatic signet ring cell carcinoma (PPSRCC), guiding clinical decision-making and improving prognosis.

**Methods:**

In addition to the narrative synthesis, we performed a targeted extraction and comparative synthesis of published patient-level cases, case series, and registry studies to generate pooled clinicopathologic summaries, propose an operational diagnostic workflow (imaging–EUS-FNA–IHC–molecular testing), and formulate PPSRCC-specific management recommendations.

**Results:**

Primary pancreatic signet ring cell carcinoma (PPSRCC) is a rare pancreatic cancer subtype with extremely high malignancy and very poor prognosis. Current research on this disease is limited with weak evidence, leading to insufficient clinical awareness. PPSRCC exhibits unique epidemiological characteristics, molecular mechanisms, and clinical diagnostic and therapeutic features. Molecular studies suggest that its carcinogenic pathways involve abnormal activation of the ErbB2/ErbB3-MUC4 axis, activation of the PI3K and MAPK pathways, and frequent mutations in classical pancreatic cancer driver genes such as KRAS; however, the lack of specific molecular biomarkers and typical imaging findings makes clinical diagnosis challenging. In terms of treatment, clinical practice mostly refers to the therapeutic strategies for pancreatic ductal adenocarcinoma (PDAC), primarily surgery combined with radiochemotherapy. Targeted therapies against KRAS mutations and immunotherapy targeting PD-L1 may provide new directions for future management. In-depth elucidation of the molecular features of PPSRCC, clarification of diagnostic and therapeutic strategies, and development of novel targeted agents are key to improving clinical outcomes.

**Conclusions:**

Deeper mechanistic interrogation and standardized diagnostic workflows are needed to enable earlier detection and tailored therapy in PPSRCC. Multidisciplinary care and emerging precision strategies may improve outcomes.

**Supplementary Information:**

The online version contains supplementary material available at 10.1186/s12957-025-04106-4.

## Background

Pancreatic cancer is a gastrointestinal malignancy with insidious onset and rapid progression. Pancreatic ductal adenocarcinoma (PDAC) is the most common histological type, accounting for approximately 85.8% of cases [1]. Primary pancreatic signet ring cell carcinoma (PPSRCC) is an exceptionally rare and more aggressive special subtype of pancreatic adenocarcinoma, representing less than 1% of pancreatic cancers, with a 5-year survival rate of only about 3.4%, much lower than the overall 5-year survival of pancreatic cancer (13%) [2–4]. This review provides a systematic and comprehensive summary of recent advances in the diagnosis and treatment of PPSRCC, aiming to improve disease recognition, optimize clinical decision-making, and ultimately enhance patient outcomes.

## Methods and materials

This is a narrative review that synthesizes peer‑reviewed studies, registry analyses, and case series on PPSRCC. Key themes include epidemiology, molecular mechanisms, diagnostic workflows, and management strategies.

### Narrative Review Methods

This review followed the methodological guidance for rigorous narrative reviews proposed by Sukhera [5]. Terminology and case definition. In accordance with the 5th edition of the WHO Classification of Tumours of the Digestive System (2019), pancreatic tumors showing signet-ring cell morphology are categorized under the broader entity of signet-ring cell (SRC)/poorly cohesive carcinoma [6]. To maintain consistency with existing literature and clinical reports, we continue to use the term primary pancreatic signet-ring cell carcinoma (PPSRCC) throughout this review, while explicitly aligning it with the WHO SRC/poorly cohesive category. Unless otherwise specified, PPSRCC herein refers to pancreatic ductal adenocarcinoma with signet-ring cells comprising ≥ 50% of the tumor and that metastasis from other organs is excluded.We conducted a comprehensive search of the literature from database inception through June 30, 2025, without language restrictions. The core Boolean search string used was: (pancreas OR pancreatic OR ampulla OR periampullary) AND (“signet ring” OR signet-ring OR “poorly cohesive” OR SRC) AND (carcinoma OR adenocarcinoma). Controlled vocabulary (MeSH/Emtree) and proximity operators were adapted for each database. We searched the following databases: PubMed, Web of Science, Scopus, and Embase, selected to ensure comprehensive coverage of the relevant literature. We included case reports, case series, registry-based analyses, and reviews reporting clinicopathologic, molecular, diagnostic, or therapeutic characteristics of pancreatic signet-ring cell carcinoma (SRC)/poorly cohesive carcinoma, in accordance with the current WHO classification. We excluded non-primary pancreatic metastases, animal studies, and non-peer-reviewed materials. Two independent reviewers screened titles, abstracts, and full texts; discrepancies were resolved by consensus. Extracted data were organized into thematic domains, including epidemiology, molecular mechanisms, diagnostic strategies, and therapeutic management, and synthesized narratively to identify shared features and knowledge gaps. Given the rarity and heterogeneity of PPSRCC, quantitative meta-analysis was not feasible, so a narrative synthesis was adopted to integrate the best available evidence and highlight future research directions.

## Results

### Epidemiology

Signet ring cell carcinoma (SRCC) can arise in multiple organs, with more than 96% of cases originating in the stomach, followed by the rectum, colon, gallbladder, bladder, breast, and pancreas [7]. As a very rare pancreatic cancer subtype, PPSRCC differs markedly from common PDAC in epidemiological features and prognosis (Table 1). According to SEER database analyses, the incidence of PPSRCC is only 0.349 per million, far lower than 10.798 per million for PDAC [8]. Regarding age at onset, both PPSRCC and PDAC occur mainly in middle-aged and older adults aged 58–72 years; approximately 50.5% of PPSRCC cases fall within this age range, slightly higher than PDAC (44.7%). With respect to race and sex, both tumors are more common in White males, and the incidence of PPSRCC is higher than that of PDAC [9]. From a disease progression perspective, at diagnosis PPSRCC is more likely to present with lymph node metastasis (38.8% vs. 35.3%) and distant metastasis (69.4% vs. 52.0%), suggesting stronger aggressiveness [8, 10]. In TNM staging, 62.4% of PPSRCC patients are already stage IV at diagnosis, much higher than PDAC (51.6%),while the proportions of stages I and II are significantly lower, indicating poorer early detection and more advanced disease at presentation. Consequently, the proportions undergoing surgery and chemotherapy are both lower in PPSRCC than in PDAC. Prognostically, survival in PPSRCC is significantly worse than in PDAC: 1-, 3-, and 5-year overall survival (OS) rates are 19.67%, 4.96%, and 3.01%, respectively, versus 28.33%, 7.67%, and 4.61% for PDAC; median OS is only 3 months for PPSRCC compared with 6 months for PDAC [9, 10]. These findings reflect the diagnostic difficulty, rapid progression, poor treatment response, and extremely poor prognosis of PPSRCC, warranting high clinical vigilance.

**Table 1:** Clinical characteristics of pancreatic signet ring cell carcinoma versus pancreatic ductal adenocarcinoma [8–10]

Variable	PPSRCC	PDAC
Incidence (per million)	0.349	10.798
Peak age (years)	58–72(50.5%)	58–72(44.7%)
Predominant sex	Male (59.7%)	Male (51.7%)
Common race	White (82.2%)	White (79.7%)
Lymph node metastasis rate	38.8%	35.3%
Distant metastasis rate	69.4%	52%
TNM stage—Stage I	2.4%	6.3%
TNM stage—Stage II	23.1%	26.4%
TNM stage—Stage III	7.1%	9.7%
TNM stage—Stage IV	62.4%	51.6%
Chemotherapy	47.7%	57.1%
Surgery	7.4%	8.4%
1-year OS	19.67%	28.33%
3-year OS	4.96%	7.67%
5-year OS	3.01%	4.61%
Median OS (months)	3	6

### Carcinogenic mechanisms

**Fig. 1:**
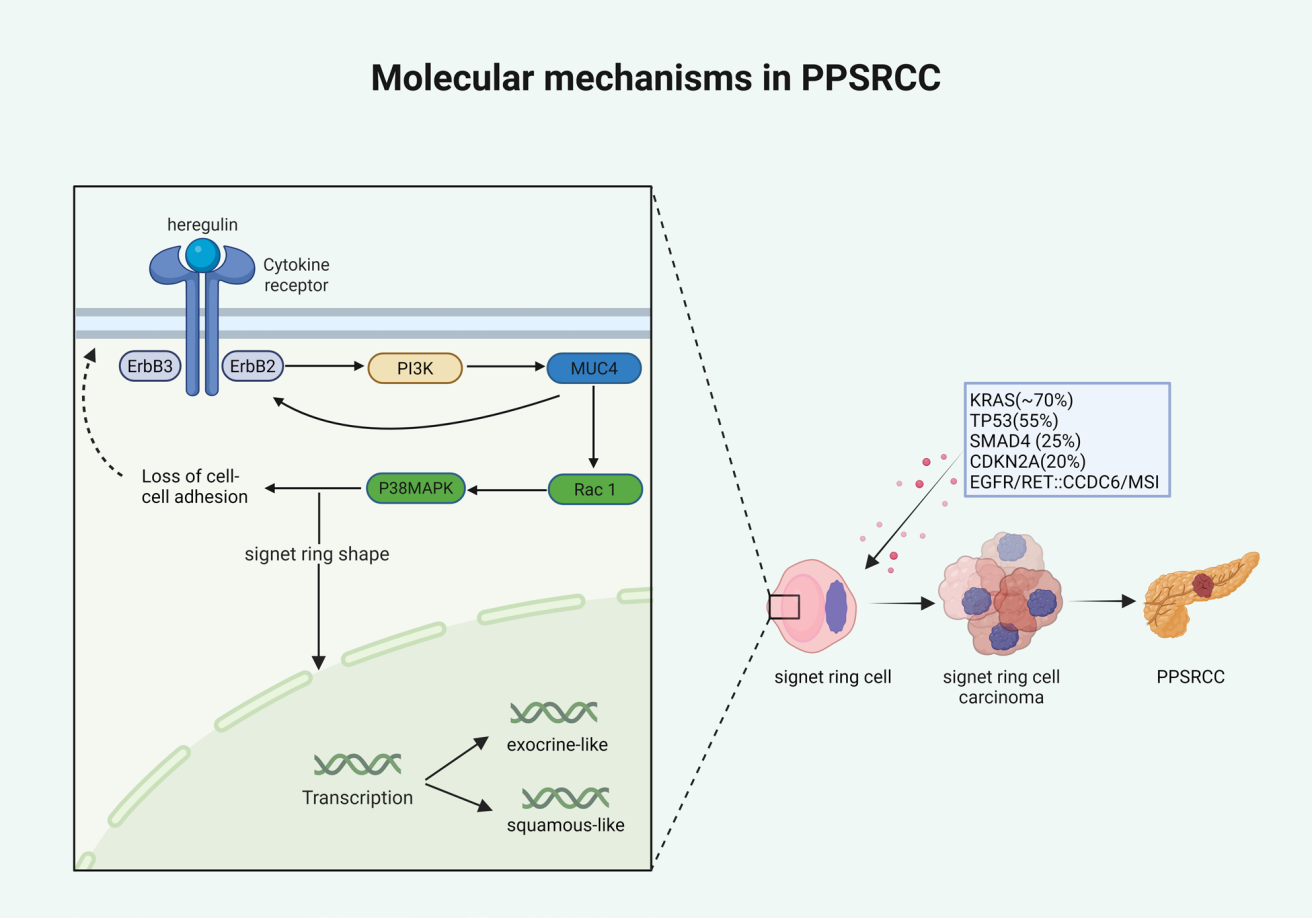
Proposed mechanisms underlying PPSRCC formation(Created in BioRender. jie, z. (2025) https://BioRender.com/06rg19h) [12–14]

PPSRCC is a rare mucin-producing variant of pancreatic adenocarcinoma with an unclear cell of origin. While some reports suggest a lamina propria stem-cell origin [9], this has not been substantiated in PPSRCC pathological studies [11]. PPSRCC cells contain abundant intracellular mucin, which pushes the cell nucleus to the periphery, forming a characteristic ‘signet ring’ morphology. Despite its histological resemblance to gastric signet ring cell carcinoma, there are differences in molecular carcinogenic mechanisms. Current research indicates that the pathogenesis of PPSRCC is closely related to the sustained activation of the ErbB2 (tyrosine kinase receptor 2)/ErbB3 (human epidermal growth factor receptor 3) signaling complex (Fig. 1) [12]. This complex stimulates the PI3K pathway, thereby enhancing the secretion of MUC4 mucin within tumor cells; MUC4, in turn, activates ErbB2, forming a sustained positive feedback loop involving the ErbB2/ErbB3-MUC4 axis. This signaling axis not only promotes the formation of the typical signet ring morphology but also disrupts cell-cell adhesion by activating the Rac1 and p38 MAPK pathways, inducing a transition to a low-adhesion, highly invasive phenotype.

### Molecular features

At the genomic level, PPSRCC shares high similarity with PDAC, with a mutational spectrum mainly involving classical pancreatic cancer driver genes such as KRAS (~ 70%), TP53 (55%), SMAD4 (25%), and CDKN2A (20%). In addition, individual cases have shown EGFR mutations, RET::CCDC6 fusion, and microsatellite instability (MSI), providing potential molecular targets for precision therapy [13].Notably, the classic SRCC driver gene CDH1 in gastric SRCC is not mutated in PPSRCC, indicating a marked genetic distinction from gastric SRCC. Transcriptomic analyses suggest two molecular subtypes—exocrine-like and squamous-like—similar to the subtype distribution in PDAC, implying biological heterogeneity within PPSRCC. More broadly, Schneider et al. emphasized the importance of tissue-specific tumorigenesis: although multiple cancers may share similar driver mutations (e.g., KRAS, TP53), the phenotypic consequences differ among tissues due to variations in microenvironment, cell of origin, and signaling context. PPSRCC exemplifies this tissue-specific background [14].Therefore, PPSRCC arises through a complex, multi-pathway process involving loss of intercellular junctions, mucin overproduction, and driver mutations. The associated pathways and molecular targets provide an important basis for future molecular classification, prognostic assessment, and targeted therapy.

### Clinical features

PPSRCC shares clinical symptoms with pancreatic cancer, most commonly epigastric discomfort, dull pain, dyspepsia, and jaundice. Due to its insidious onset, initial symptoms are closely related to tumor location and extent [15]. Among primary sites, intraductal tumors are more likely to present first with obstructive jaundice, followed by tumors in the pancreatic head; SRCCs in the body and tail often lack typical early symptoms and, by the time abdominal pain prompts medical attention, invasion of adjacent organs or distant metastasis is common [16].

### Tumor biomarkers

CA19-9 is the most valuable laboratory indicator for pancreatic cancer diagnosis [17]. However, in an analysis of 11 PPSRCC cases by Daniel et al., only four patients had elevated CA19-9 and three had elevated CEA. There are currently no highly sensitive tumor markers to aid in the early diagnosis of PPSRCC [18].

### Imaging features

Given its favorable spatial and temporal resolution, CT is currently the best noninvasive imaging modality for evaluating the pancreas [15]. Early PPSRCC on CT resembles pancreatic adenocarcinoma, typically presenting as a focal, heterogeneous low-attenuation lesion [19]. It should be noted that atypical CT findings mimicking chronic pancreatitis can also occur [20]. When diagnosis remains uncertain or contrast-enhanced CT is contraindicated, enhanced MRI can be used for further differentiation. For pancreatic malignancies with hepatic metastases, MRI is more sensitive than CT, with pooled positivity rates of 83% (95% CI 74–88) versus 45% (95% CI 21–71) for CT [21].

### Pathology and diagnostic criteria

When noninvasive tests are inconclusive, a combination of endoscopic ultrasonography (EUS), endoscopic retrograde cholangiopancreatography (ERCP), and EUS-guided fine-needle aspiration (EUS-FNA) can be considered. However, EUS-guided biopsy or ERCP-guided pancreatic duct biopsy can still yield false-negative results [22]. Thus, the classical diagnostic criterion for PPSRCC remains identification of typical signet ring cells constituting >50% of adequately sampled tissue on hematoxylin-eosin staining.

### New diagnostic strategies (MDT and AI)

For difficult cases, a multidisciplinary team (MDT) model can be considered. The MDT model at Zhongshan Hospital, Fudan University, has been shown to integrate resources across specialties to achieve more rapid and accurate diagnosis of complex pancreatic tumors, formulate rational treatment plans, and improve postoperative survival in patients with pancreatic malignancies [23].With the rapid development of artificial intelligence (AI), its value in the early diagnosis of pancreatic malignancies is increasingly evident [24].Deep learning-based imaging analysis can identify subtle pancreatic lesions on CT/MRI that are difficult to detect by the naked eye and achieves higher accuracy than conventional methods [25, 26].Liquid biopsy combined with AI algorithms may enhance ultra-early capture of circulating tumor cells [27].Furthermore, AI is important for early identification of high-risk populations. Studies from the United States and Denmark have demonstrated that machine and deep learning models based on electronic health records can identify individuals at high risk of PDAC 6–18 months in advance, with the best models predicting up to 36 months earlier [28, 29].These technologies not only improve diagnostic efficiency but also provide new avenues to overcome bottlenecks in traditional diagnosis.

### Differential diagnosis

In differential diagnosis, PPSRCC should primarily be distinguished from other primary pancreatic tumors and metastatic SRCC. Differentiation from primary pancreatic tumors is mainly based on microscopic cytomorphology. However, when certain pancreatic tumors exhibit signet-ring-like cellular features that make histological distinction difficult, immunohistochemical (IHC) staining is required (Table 2). Siba et al. [10].reported that different pancreatic tumors display distinct IHC expression profiles: PPSRCC is positive for MUC1 and MUC5; primary pancreatic clear cell carcinoma is positive for HNF1B, PAS, and PAS-D; pancreatic colloid carcinoma is positive for CDX2 and MUC2; vacuolated pancreatic adenocarcinoma is positive for CA19-9 and MUC1, partially positive for CEA and B72.3, positive for CK34βE12 (54.5%), and negative or only focally positive for MUC2 [30]; acinar cell carcinoma shows positivity for CEA, MUC1, and CK8, mainly localized to brush border–like regions; pancreatic neuroendocrine tumors with signet-ring cell features are positive for synaptophysin and chromogranin; and solid pseudopapillary neoplasms with signet-ring cell features are positive for β-catenin and CD10. In contrast, differentiation from metastatic SRCC requires comprehensive imaging studies such as CT or PET-CT to exclude metastases from other organs. For SRCC of unknown origin, mucin profiling may help determine the primary source of the metastasis [31].There is another point to make, before establishing a diagnosis of PPSRCC, secondary involvement by ampullary signet-ring cell carcinoma (ASRCC) must be carefully excluded. Although ASRCC is still rare, it tends to grow aggressively and frequently invades the pancreatic head or pancreatic duct [32]. Therefore, evaluation of the ampullary region and exclusion of secondary invasion require comprehensive cross-sectional imaging (CT/MRI) and, when appropriate, endoscopic assessment (ERCP or EUS) [33]. Moreover, ampullary SRCC can be subclassified by immunohistochemical (IHC) staining patterns into intestinal (I type), pancreatobiliary (PB type), gastric, and mixed types (Table 3) [34]. Histologically, ASRCC may display either intestinal or pancreatobiliary differentiation: the pancreatobiliary type, usually arising from distal pancreatic or biliary epithelium, is typically positive for CK7, CK19, and MUC1 but negative for CK20 and CDX2; the intestinal type, originating from the ampullary intestinal mucosa, shows the opposite pattern (CK20+, MUC2+, CDX2+, CK7–) [35]. Recognition of these phenotypic features, together with careful histomorphologic assessment, is essential for distinguishing morphologically similar pancreatic tumors, determining an ampullary origin, and avoiding misclassification of ASRCC with pancreatic extension as primary PPSRCC.

**Table 2:** Differential diagnosis between PPSRCC and pancreatic tumors with signet ring-like morphology[10, 30, 31]

Tumor Type	Main Immunohistochemical Markers	Diagnostic Highlights
Primary pancreatic signet-ring cell carcinoma	MUC1, MUC5 positive	Signet-ring cell carcinoma originating in the pancreas
Primary pancreatic clear cell carcinoma	HNF1B, PAS, PAS-D, pankeratin, CK7, EMA, CEA positive; CK20, HMB45, and neuroendocrine markers (e.g., Synaptophysin, Chromogranin) negative	Clear cytoplasm, positive glycogen staining, negative MUC
Pancreatic colloid carcinoma (CC)	CDX2, MUC2, CEA (basal + apical membrane) positive; low CA19-9 expression	Abundant mucin, intestinal-type differentiation, mucin nodule–like infiltration pattern
Vacuolated pancreatic carcinoma	CA19-9, MUC1 positive; CEA, B72.3 partially positive; CK34βE12 positive (54.5%); MUC2 negative or only focally positive	Cells with marked vacuolation, arranged in cohesive nested patterns
Acinar cell carcinoma	CEA, MUC1, CK8 positive (mainly along brush border-like areas)	Brush border structure, foamy vacuolated cytoplasm, positive acidic mucin staining
Pancreatic neuroendocrine tumor with signet-ring cell features	Synaptophysin, Chromogranin positive	No mucin positivity, nuclear displacement due to myelin body accumulation, neuroendocrine differentiation
Solid pseudopapillary neoplasm (SPPN) with signet-ring cell features	β-catenin, CD10 positive; PAS, mucicarmine, and Oil Red O negative	Multivacuolated clear cells without mucin; vacuoles formed by dilated mitochondria and smooth endoplasmic reticulum
Metastatic signet-ring cell carcinoma	Variable expression; requires correlation with imaging and mucin profile	Exclude primary tumors from stomach, colorectum, and other organs

**Table 3:** Differential diagnosis of ampullary SRCC[34, 35]

Tumor Type	Main Immunohistochemical Markers	Diagnostic Highlights
Ampullary SRCC-intestinal type (I-type)	CK20 positive, MUC2 positive, CDX2 positive; CK7 negative	Originates from the intestinal mucosa of the ampulla
Ampullary SRCC-pancreatobiliary type (PB-type)	CK7 positive, CK19 positive, MUC1 positive; CK20, CDX2 negative	Originates from the distal pancreatic or biliary duct epithelium
Ampullary SRCC-gastric type	MUC5AC, MUC6 positive	Gastric-type differentiation, may coexist with intestinal and pancreatobiliary types
Ampullary SRCC-mixed type	Co-expression of intestinal-type markers (MUC2, CDX2) and pancreatobiliary-type markers (MUC1, CK7)	Derived from both intestinal and pancreatobiliary epithelium, with mixed differentiation

### Treatment and prognosis

PPSRCC is rare and lacks dedicated guidelines; current management largely follows strategies for pancreatic adenocarcinoma. Main modalities include surgery, chemotherapy, radiotherapy, and combined approaches; targeted and immune therapies remain exploratory.

### Surgery

Surgical resection is the only curative modality for PPSRCC and is generally the first-line option for resectable or locally advanced pancreatic tumors [36]. For resectable malignant tumors in the pancreatic head, pancreaticoduodenectomy remains the classic preferred procedure, and R0 resection requires seven negative margins with adequate lymphadenectomy and retrieval of at least 16 lymph nodes [37]. For resectable body-tail tumors, radical antegrade modular pancreatosplenectomy (RAMPS) is increasingly recognized. Emphasizing deeper dissection planes, antegrade, modular, and en bloc resection principles and standardized lymphadenectomy, RAMPS better aligns with the “no-touch” concept, increases R0 rates, and shows advantages in postoperative local control [38]; the ESMO clinical practice guideline recommends RAMPS for body-tail tumors [37]. For borderline resectable and locally advanced disease, the latest NCCN guideline underscores the necessity of neoadjuvant chemotherapy [39]; the latter in particular does not benefit from upfront surgery, and selective resection after active preoperative therapy is recommended to achieve better long-term outcomes [40]. Notably, a new three-dimensional restaging principle—the Anatomy—Biology—Condition (ABC) nomenclature—has been emphasized, advocating preoperative assessment of resectability by integrating imaging features, CA19-9, and general condition to optimize surgical decision-making [41–45].

### Chemotherapy

Surgery is usually recommended for non-metastatic PSRCC, whereas chemotherapy is the first choice for metastatic disease [10]. Although SRCC is generally less chemosensitive, appropriate regimens can still improve outcomes in PPSRCC [46]. Only limited reports are available regarding specific regimens. A retrospective cohort study indicated that postoperative chemotherapy significantly prolonged median survival (16 vs. 10 months), although no head-to-head comparisons of regimens were conducted [47]. In a case reported by Radojkovic et al., single-agent gemcitabine was used due to low creatinine clearance; the tumor shrank from 4.5 cm to 1.5 cm, enabling resection [48]. Nakamura et al. described multivisceral resection followed by oral 5-fluorouracil initiated 1 month postoperatively and continued for 1 year, with no recurrence at 16-month follow-up [49]. A rare case of pancreatic body adenocarcinoma with concomitant tail SRCC was reported, in which the patient underwent radical resection followed by 6 months of modified FOLFIRINOX (oxaliplatin, irinotecan, leucovorin, and 5-fluorouracil), and remained recurrence-free at 11 months postoperatively [50]. Alberto et al. considered FOLFIRINOX, gemcitabine plus nab-paclitaxel (GEM-Nab-Pac), and nal-IRI plus 5-FU (NALIRIFOX) as relatively effective options for PPSRCC [51]. The international NAPOLI-3 trial showed longer median OS with NALIRIFOX versus GEM-Nab-Pac in pancreatic cancer (11 vs. 9 months) with manageable toxicity [52]. The optimal chemotherapy for PPSRCC remains undetermined and requires higher-level evidence.

### Radiotherapy

As a local treatment, radiotherapy may improve local control and potentially increase resection rates in borderline-resectable and locally advanced pancreatic cancer [53], but its benefit in PPSRCC remains controversial. Patel et al. found that external-beam radiotherapy was an independent factor improving OS and disease-specific survival (DSS) in postoperative and metastatic PSRCC [8]. Others argue that radiotherapy alone does not improve prognosis and that benefits arise only when combined with surgery or chemotherapy [9, 47]. The “abscopal effect”has been proposed, whereby radiotherapy exerts direct local cytotoxicity while also inducing immune-mediated systemic effects that suppress tumor growth outside the radiation field, possibly benefiting distant metastases [54]. Therefore, radiotherapy may confer positive outcomes in PPSRCC through synergy between local and immune-mediated systemic effects.

### Targeted and immunotherapy

Exploration of targeted therapy in pancreatic cancer is progressing, whereas breakthroughs in immunotherapy remain limited. The latest NCCN guideline recommends larotrectinib or entrectinib as first-line therapy for KRAS-wildtype tumors harboring NTRK fusions [55, 56]. Beyond inhibitors targeting KRAS G12C, novel agents against KRAS G12D and pan-KRAS are under development with active clinical investigation [57, 58]. Genomic profiling in PDAC and PPSRCC has revealed EGFR mutations, amplification, somatic PTEN mutations, RET (exon 12),CCDC6 (exon 8) fusion, and MSI as potential targets for precision therapy.12 Moreover, Canbey et al. reported a significantly higher PD-L1 positivity rate in PPSRCC associated with worse survival, suggesting PD-L1 as a potential immunotherapeutic target [59]. Given the high prevalence of KRAS mutations in PPSRCC, KRAS-targeted therapies may also be applicable, requiring further clinical validation.

## Conclusion

PPSRCC is a highly invasive malignancy predominantly affecting older men; compared with gastrointestinal SRCC, it shows poorer treatment responses and shorter survival, posing a substantial health threat [60].Current management faces three major challenges: (1) lack of specific molecular biomarkers or imaging features hampers early diagnosis; (2) the highly invasive nature of SRCC limits the efficacy of conventional radiochemotherapy; and (3) a shortage of precise molecular targets. Present strategies largely draw on experiences from pancreatic adenocarcinoma, highlighting the need to establish a diagnostic therapeutic framework tailored to the unique biology of PPSRCC. Surgery combined with radiochemotherapy remains the most effective approach to date, with particular attention to negative margins and thorough lymphadenectomy. To overcome current bottlenecks, future research should focus on: (1) deeper mechanistic dissection of tumorigenesis and invasion using single-cell and other omics technologies; (2) further identification of actionable targets within PPSRCC pathogenesis; and (3) development of KRAS-related targeted drugs. With the integration of precision medicine and AI technologies, the management of PPSRCC is expected to transition from experience-based treatment to molecular subtype-guided personalized therapy, ultimately improving patient outcomes.

## Supplementary Information

Supplementary Material 1.

Supplementary Material 2.

Supplementary Material 3.

## Data Availability

No new data were generated or analysed in this study.

## Abbreviations

PPSRCC: Primary pancreatic signet ring cell carcinoma
PDAC: Pancreatic ductal adenocarcinoma
IHC: Immunohistochemistry
EUS-FNA: Endoscopic ultrasoundguided fineneedle aspiration
MDT: Multidisciplinary team
MSI: Microsatellite instability
ERCP: Endoscopic retrograde cholangiopancreatography
CT/MRI: Computed tomographyMagnetic resonance imaging
