# Supplementary material for: Primary pancreatic signet ring cell carcinoma: molecular mechanisms and advances in clinical diagnosis and treatment
Source: World J Surg Oncol. 2025 Nov 27;24:9. doi: 10.1186/s12957-025-04106-4 (PMC12764020; doi:10.1186/s12957-025-04106-4)
Supplement: Supplementary file 1 — Supplementary Material 1. [file 12957_2025_4106_MOESM1_ESM.zip › Table-Modified.docx]

Table 1. Clinical characteristics of pancreatic signet ring cell carcinoma versus pancreatic ductal adenocarcinoma[8-10]

| **Variable** | **PPSRCC** | **PDAC** |
| --- | --- | --- |
| Incidence (per million) | 0.349 | 10.798 |
| Peak age (years) | 58-72（50.5%） | 58-72（44.7%） |
| Predominant sex | Male (59.7%) | Male (51.7%) |
| Common race | White (82.2%) | White (79.7%) |
| Lymph node metastasis rate | 38.8% | 35.3% |
| Distant metastasis rate | 69.4% | 52% |
| TNM stage—Stage I | 2.4% | 6.3% |
| TNM stage—Stage II | 23.1% | 26.4% |
| TNM stage—Stage III | 7.1% | 9.7% |
| TNM stage—Stage IV | 62.4% | 51.6% |
| Chemotherapy | 47.7% | 57.1% |
| Surgery | 7.4% | 8.4% |
| 1-year OS | 19.67% | 28.33% |
| 3-year OS | 4.96% | 7.67% |
| 5-year OS | 3.01% | 4.61% |
| Median OS (months) | 3 | 6 |

Table 2. Differential diagnosis between PPSRCC and pancreatic tumors with signet ring-like morphology[ 10,30,31]

| **Tumor Type** | **Main Immunohistochemical Markers** | **Diagnostic Highlights** |
| --- | --- | --- |
| Primary pancreatic signet-ring cell carcinoma | MUC1, MUC5 positive | Signet-ring cell carcinoma originating in the pancreas |
| Primary pancreatic clear cell carcinoma | HNF1B, PAS, PAS-D, pankeratin, CK7, EMA, CEA positive; CK20, HMB45, and neuroendocrine markers (e.g., Synaptophysin, Chromogranin) negative | Clear cytoplasm, positive glycogen staining, negative MUC |
| Pancreatic colloid carcinoma (CC) | CDX2, MUC2, CEA (basal + apical membrane) positive; low CA19-9 expression | Abundant mucin, intestinal-type differentiation, mucin nodule–like infiltration pattern |
| Vacuolated pancreatic carcinoma | CA19-9, MUC1 positive; CEA, B72.3 partially positive; CK34βE12 positive (54.5%); MUC2 negative or only focally positive | Cells with marked vacuolation, arranged in cohesive nested patterns |
| Acinar cell carcinoma | CEA, MUC1, CK8 positive (mainly along brush border-like areas) | Brush border structure, foamy vacuolated cytoplasm, positive acidic mucin staining |
| Pancreatic neuroendocrine tumor with signet-ring cell features | Synaptophysin, Chromogranin positive | No mucin positivity, nuclear displacement due to myelin body accumulation, neuroendocrine differentiation |
| Solid pseudopapillary neoplasm (SPPN) with signet-ring cell features | β-catenin, CD10 positive; PAS, mucicarmine, and Oil Red O negative | Multivacuolated clear cells without mucin; vacuoles formed by dilated mitochondria and smooth endoplasmic reticulum |
| Metastatic signet-ring cell carcinoma | Variable expression; requires correlation with imaging and mucin profile | Exclude primary tumors from stomach, colorectum, and other organs |

Table 3. Differential diagnosis of ampullary SRCC[ 34,35]

| **Tumor Type** | **Main Immunohistochemical Markers** | **Diagnostic Highlights** |
| --- | --- | --- |
| Ampullary SRCC-intestinal type (I-type) | CK20 positive, MUC2 positive, CDX2 positive; CK7 negative | Originates from the intestinal mucosa of the ampulla |
| Ampullary SRCC-pancreatobiliary type (PB-type) | CK7 positive, CK19 positive, MUC1 positive; CK20, CDX2 negative | Originates from the distal pancreatic or biliary duct epithelium |
| Ampullary SRCC-gastric type | MUC5AC, MUC6 positive | Gastric-type differentiation, may coexist with intestinal and pancreatobiliary types |
| Ampullary SRCC-mixed type | Co-expression of intestinal-type markers (MUC2, CDX2) and pancreatobiliary-type markers (MUC1, CK7) | Derived from both intestinal and pancreatobiliary epithelium, with mixed differentiation |
