# Supplementary figures and images for: Primary pancreatic signet ring cell carcinoma: molecular mechanisms and advances in clinical diagnosis and treatment
Source: World J Surg Oncol. 2025 Nov 27;24:9. doi: 10.1186/s12957-025-04106-4 (PMC12764020; doi:10.1186/s12957-025-04106-4)

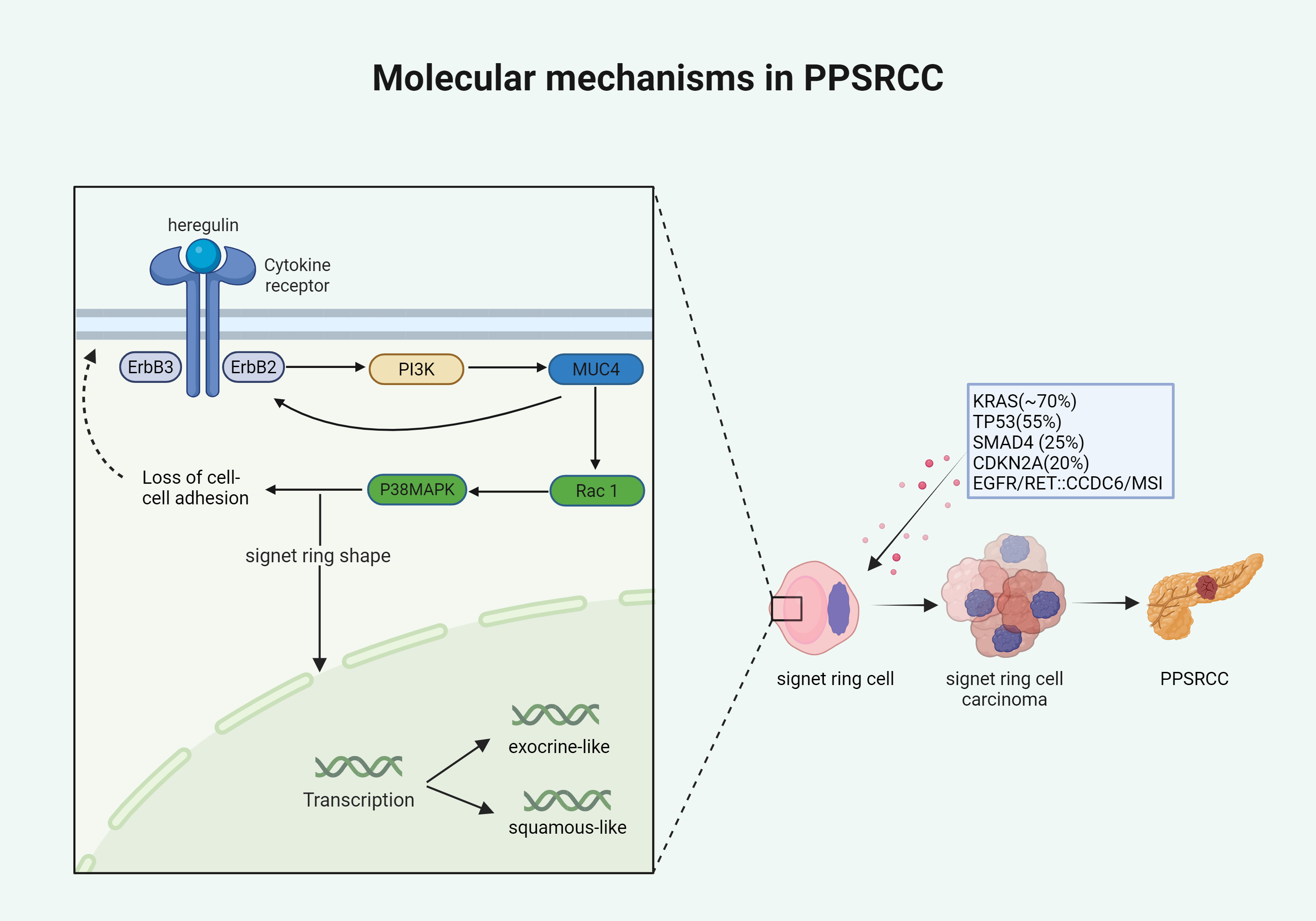

Supplement: Supplementary file 2 — Supplementary Material 2. [file 12957_2025_4106_MOESM2_ESM.zip › Molecular mechanisms in PPSRCC(300dpi)-Modified.png]
